# Effect of fuel choice on conductivity and morphological properties of samarium doped ceria electrolytes for IT-SOFC

**DOI:** 10.3906/kim-2104-56

**Published:** 2021-10-19

**Authors:** Burcu AYGÜN, Vedat SARIBOĞA, Mehmet Ali Faruk ÖKSÜZÖMER

**Affiliations:** 1 Mechanical Engineering Department, Engineering Faculty, Özyeğin University, İstanbul Turkey; 2 Chemical Engineering Department, Engineering Faculty, İstanbul University-Cerrahpaşa, İstanbul Turkey

**Keywords:** Ionic conductivity, Sol-gel processes, Samaria-doped-CeO_2_.

## Abstract

The present investigation is emphasized on the effect of various combustion agents on the crystal properties, surface microstructure, and oxygen ion conductivity of 20% mole-Sm doped ceria (Ce_0.80_Sm_0.20_O_1.90_/SDC20) ceramics as solid electrolyte for IT-SOFCs. The most widely used combustion agents for engineering ceramic production as ethylene glycol, diethylene glycol, triethylene glycol, L-alanine, L-valine, glycine, citric acid monohydrate, urea, and EDTA-citric acid were compared in terms of SDC20 properties. X-ray diffraction (XRD), scanning electron microscopy (SEM), thermogravimetric analysis (TGA), and electrochemical impedance spectroscopy (EIS) were used to determine the microstructure properties, crystal structure and ionic conductivity of SDC20 powder. XRD pattern of the ceramics revealed the formation of single-phase fluorite structure. According to the results of electrochemical analysis, the maximum total ionic conductivity was observed in SDC20 electrolyte synthesized using triethylene glycol as the fuel among all the synthesized electrolytes (5.72 x 10^–2^ S.cm^–1^).

## 1. Introduction

Solid oxide fuel cell (SOFC) is one of the energy sources that chemical energy directly converted into electrical energy with its advantages of high efficiency, fuel flexibility, and relatively low cost. Conventional solid oxide fuel cells basically consist of three components: the electronic conductor and the porous cathode and anode, and an ion-conducting electrolyte. Among the components, the electrolyte is one of most important components in a well obtaining performance since it eases the transition of oxygen ions from the anode to the cathode. In the literature, YSZ (yttrium stabilized zirconia) is the most commonly used as a commercial electrolyte for SOFC applications at the high operation temperature. In the literature, in order to lower the operating temperature of SOFCs and to synthesize an electrolyte with high oxide ion conductivity, thermal stability, and chemical compatibility over a wide temperature range, researchers investigate doped lanthanum gallate and other doped ceramic electrolytes such as cerium oxide (SDC-samarium doped ceria, GDC-gadolinia doped ceria or NDC-neodymium doped ceria) [1]. Among these structures, as cerium oxide that has the capacity to accept different dopant in solid solution, it has demonstrated to have more ionic conductivity at intermediate operation temperature (~800 °C) and a potential candidate to replace the commercial YSZ as the electrolyte for SOFCs. In this respect, alkaline earth (Ca^2+^, Sr^2+^) [2], rare earth (Gd^3+^, Sm^3+^, Nd^3+^) [3–5], and transition metal (Cu^2+^, Fe^2+^) [6] doped CeO_2_ based solid electrolytes that exhibit high ionic conductivity at intermediate operation temperatures as compared to YSZ (8% mole yttria stabilized zirconia. By adding dopant (Sm^3+^, Ca^2+^, Mg^2+^, Sr^2+^), instead of Ce^4+^ ions, the crystal structure of the materials is changed to improve the ionic conductivity properties of the electrolyte as well as to increase the ionic conductivity. In the literature, most of the studies are carried out on Sm-doped ceria electrolytes (especially 20% Sm-doped ceria electrolytes) among single-doped ceria electrolytes. The low association enthalpy between dopant cations and oxygen vacancies and high conductivity of Sm-doped cerium electrolytes provides that Sm-doped electrolytes are more promising for future applications among single dopant electrolytes [7,8]. 

In the literature, it has been reported that the sample with 20% mole Sm-doped Ceria (Ce_0.80_Sm_0.20_O_1.90 _/SDC20) prepared by various combustion methods among single doped ceria electrolytes has higher ionic conductivity than other samples [9]. There are many combustion agents applied for the preparation of SDC20 [10–15]. Interestingly, in each study the researchers claim that their proposed fuel in the relevant study is superior. In addition, although the same fuel is used, different conductivity values are determined numerically in different studies. So it is not possible to make a clear comparison between these works since the test systems, test method, other intermediate steps in the preparation process are have effect over the conductivity performance. Eventually, the answer of the question of “which is the best fuel for the preparation of SDC20 according to the oxygen-ion conductivity” is not clear to the best of our knowledge. With this motivation, ethylene glycol (EG) [10], diethylene glycol (DEG), triethylene glycol (TREG), L-alanine, L-valine, glycine [11,12], citric acid (CA) monohydrate [13], urea [14], and EDTA-citric acid (EDTA-CA) [15], which are most widely used as a combustion agents for ceramic production in the literature were selected as fuels in this study. 

Therefore, this work is a reference study in that it enables more clear comparison of fuels used on ionic conductivity and the objective of present investigation is to work the effect of various aforementioned fuels in the preparation of Ce_0.80_Sm_0.20_O_1.90_ (SDC20) materials by the combustion method. Also ethylene glycol (EG), diethylene glycol (DEG), triethylene glycol (TREG) fuels were used for the first time as combustion agents for the preparation of SDC20 electrolytes. In addition, alanine and valine fuels have been deeply studied for the first time. Furthermore, there is no study in the literature comparing such a large number (9) of fuels, especially solid oxide fuel cell electrolyte synthesis. Physicochemical properties of SDC20’s synthesized using the aforementioned fuels have been revealed and the most useful fuel in terms of oxygen ion conductivity performance has been pointed. 

## 2. Experimental

### 2.1. Preparation of SDC20 powders and pellets

Ce_0.80_Sm_0.20_O_1.90_ powders were fabricated using the well-known combustion procedure. All raw materials used to fabricate desired electrolytes were: highly pure cerium (III) nitrate hexahydrate Ce(NO_3_)_3_·6H_2_O, 99.99% (Aldrich), samarium (III) nitrate hexahydrate (Sm(NO_3_)_3_·6H_2_O), 99.99% (Aldrich), ethylene glycol (C_2_H_6_O_2_), 99.99% (Aldrich), diethylene glycol (C_4_H_10_O_3_), 99.99% (Aldrich), triethylene glycol (C_6_H_14_O_4_), 99.99% (Aldrich), L-alanine (C_3_H_7_NO_2_), 99.5% (Aldrich), L-valine (C_5_H_11_NO_2_), 99.5% (Aldrich), glycine (C_5_H_11_NO_2_), 99.7% (Aldrich), citric acid monohydrate (C_6_H_8_O_7_.H_2_O), 99.5% (Aldrich), urea (CH_4_N_2_O), 99.5% (Aldrich), and EDTA (C_10_H_16_N_2_O_8_, Merck). 

The amount of nitrate salts of the reactants required to produce 1 mole of SDC20 was calculated based on the propellant chemistry [16]. 

0.8Ce (NO_3_)_3 _+ 0.2Sm (NO_3_)_3 _+ 0.79C_6_H_8_O_7_ = SDC20 + 4.73CO_2_ + 1.50N_2 _+ 3.16H_2_O.

**Figure 1:**
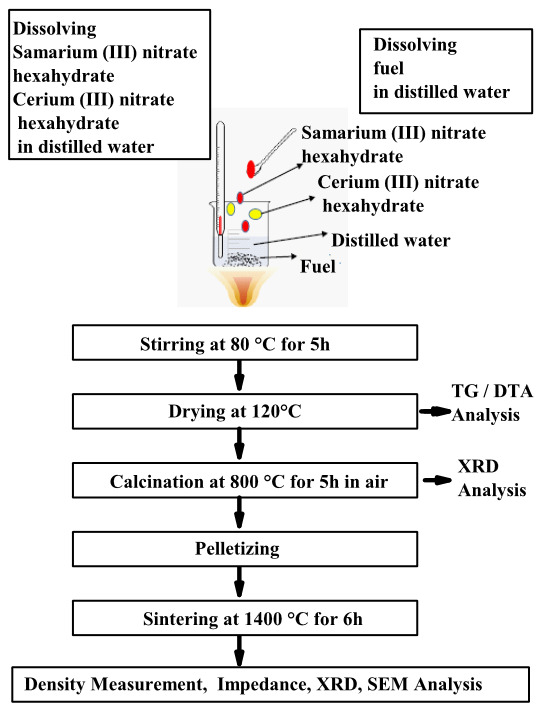
Flow chart of the combustion synthesis process.

A representative reaction for the citrate-nitrate method is given above. The reactions occurring in all combustion processes are similar. Total amount of gases produced per one mole formation of samarium doped ceria in the combustion reactions used other fuels were given in Table 1. As can be seen, for citrate-nitrate combustion method this value is calculated by the mole number of gas phase products as ≈ 9.4 (CO_2_+N_2_+H_2_O). For synthesis of SDC20, cerium nitrate hexahydrate, the corresponding amount of samarium (III) nitrate hexahydrate and the fuel in stoichiometric ratio were dissolved in distilled water separately. Ethylene glycol, diethylene glycol, triethylene glycol, glycine, L-valine, L-alanine, citric acid, urea, and EDTA were used as fuel. Then, the solution was heated to 80 ºC and mixed until the water in the solution evaporated and gel formation was observed. Auto-combustion happened after gel formation and yellow SDC20 in fine powder structure was obtained. After calcination at 800 °C for 5 h with a heating rate of 5 °C.min^–1^, the oxide powders were confirmed by XRD analysis to be single phase and of fluorite structure. The Ce_0.80_Sm_0.20_O_1.90_ “ashes” were pressed at a pressure of about 200 MPa by cold isostatic press to obtain compact cylindrical pellets. Then the electrolytes were sintered in static air at 1400 °C for 6 h with a heating rate of 5 °C.min^–1^ to form a dense electrolyte. The flow chart of the combustion process is given in Figure 1*.*

**Table 1:** Effect of fuels on adiabatic flame temperature, the enthalpy of reaction and the total amount of gases produced from the nine combustion reactions per 1 mole SDC20.

Fuel	Tadiabatic (K)	ΔHr (kJ/mol)	Total gas products *(mol)
Glycine	2845	–1224	9.4
L-Alanine	3198	–1254	8.1
L-Valine	3507	–1272	7.3
EG	3635	–1363	8.6
DEG	3680	–1432	7.9
TREG	3695	–1444	7.4
CA	2830	–1269	9.4
EDTA-CA(1:1)	3134	–1305	8.6
Urea	5854	–1180	13.3

* The total amount of gases produced per 1 mole SDC20 production.

### 2.2. Characterization of the powders and pellets

Thermal behaviors of the powders were ascertained after the pre-drying at 80 °C by thermogravimetric analysis by using the SII Exstar 6000 TG/DTA 6300 analyzer. TG analysis was carried out in an air atmosphere from room temperature to 350 ºC with a heating rate of 2 ºC/min and a heating rate of 10 ºC/min from 350 ºC to 1000 ºC. Structural characterization of the ceramics were carried out by Rigaku D/max-2200 ultima X-ray diffractometer with CuKα radiation (λ: 1.5406 Å). The diffractometer was operated at 40 kV and 35 mA at a scanning range of 10–90 °, with a step size of 2 º. Crystallite size of the calcined and sintered powder of Sm doped ceria, was determined from X-ray line broadening analysis. The crystallite size of the calcined and sintered powder was determined by using the well-known Scherrer’s formula: (1) where, *D*, *λ*, *β*, and *θ* are the average crystallite size (nm), the wavelength of X-ray (1.5406 Å), the full width at half maxima of the peak (FWHM) and the scattering angle of the main reflection (111), respectively.

(1)D=0.9ʎ𝛽cosθ

The lattice parameters of all samples were determined by using Bragg’s law and the following formula:

(2)a=h2+k2+l2

(3)d=ʎ2sinθ

where,

*a*: lattice parameter,

*d*: the planar spacing,

*h*, *k* and *l* are the Miller indices of the plane.

Scanning electron microscope (SEM, FEI Quanta FEG 450) was used for microstructure characterization of powder and sintered pellets. To gather high-quality images from a SEM, all electrolytes were coated with Au/Pt. 

To measure O^2–^ ionic conductivities of the pellets, Ag paste and leads were used on both sides of the pellets as electrode material (anode and cathode) followed by baking at 800 °C for 20 min. The ionic conductivities of electrolytes were determined between 300 and 800 °C with 50 °C intervals under static air by using an SOLARTRON 1260 FRA and 1296 interface in the frequency range from 10 mHz to 30 MHz with a 10 mV. Data of these structures was collected and fitted to the corresponding equivalent circuits and the Nyquist plots obtained by using a SMART program and ZSimpWin software respectively. 

## 3. Results and discussion

### 3.1. Thermal, microstructure, and phase analysis

In this study, the effects of nine different combustion agents on the properties of electrolyte materials were investigated. One of the parameters that affect the properties of the electrolyte material is the adiabatic flame temperature of the fuel. The flame temperature depends on the chemical structure of the combustion agent. The enthalpy of the combustion reaction was given as following:

(5)∆rHm0=∑B(yb∆fHm0

where *Y* *_b_* is the stoichiometric coefficient and is formation enthalpy of each reactants and products. As can be seen in Eq.5., in order to calculate the reaction enthalpy (, it is needed to look up the standard formation enthalpies ( for each of the reactant and product species involved in the combustion reactions [17,18]. The adiabatic flame temperatures were calculated by Eq.6.

(6)Q=-∆rHm0=∫298T∑B(ybcp,m)productsdT

where *Q* is the heat energy absorbed by products under adiabatic condition, and *c* *_p,m_* is the heat capacity of products at a constant pressure. Used *c* *_p,m_* data of each reactant and product were found in the literature [19]. Theoretical adiabatic flame temperature of the fuels, reaction enthalpies and the amount of total gas products for one mole SDC20 preparation were given in Table 1. 

**Figure 2:**
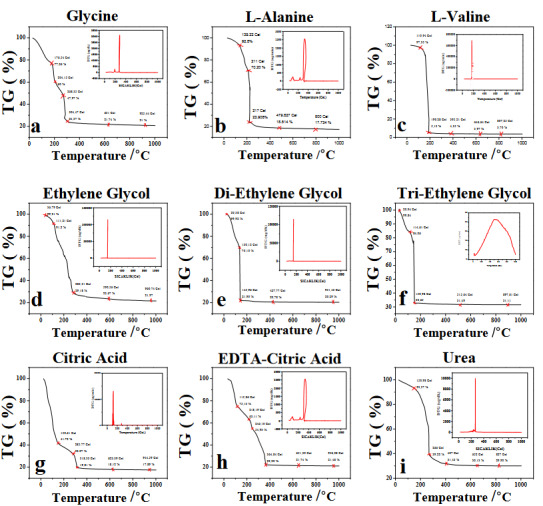
TG curves of SDC20 gels prepared with the various fuels as: a) Glycine, b) L-alanine, c) L-valine d) EG, e) DEG, f ) TREG, g) CA, h) EDTA-CA, and i) CA with a heating rate of 10 °C/min.

Figure 2 shows TG plots of the as-synthesized SDC20 powders. TG graphs of the samples show generally three distinct steps. The first step (40–150 °C) is attributed to the removal of physisorbed moisture on the surface of SDC20 particles. For the sample prepared with glycine as a fuel (Figure 2a), TG analysis shows that most of the organic substances in the structure are removed at temperatures lower than 600 °C. Below 600 °C, weight loss is around 230–250 °C due to complex decomposition reactions of organic residue [20]. Similarly, from Figures 2b, 2c, 2e, and 2f, the sharp decrease in the weight observed in the second step is determined (150–210 °C) while in Figure 2d, it is seen that weight loss increases slowly. This weight loss could be ascribed to combustion of the precursor, and almost no weight loss could be determined after the decomposition reactions of organics 400 °C ≤ [10]. Data obtained from Figure 2g shows that weight loss of the SDC20 powder precursor mainly determined below 310 °C. This means that main physical and chemical changes occurred below 310 °C. The weight loss around 150 °C and 240 °C could be related to the burn-out of precursor and residual organic materials, respectively. The corresponding TG curve of the precursor that were prepared with EDTA-CA also had three weight loss steps: one at 200°C for the citrate and the other at 260 °C for the EDTA complex decomposition, however, the third weight loss at 380 °C is related to carboxyl group (Figure 2h). From Figure 2i, it can be seen that the TG curve of the as-synthesized gel and the weight loss of 70.10% from 40 to 800 °C. Weight loss continues up to approximately 700 °C. According to the TG result, the powder prepared with urea should be calcined at least 700 °C [21]. When TG graphs of all samples were evaluated, the common calcination temperature was chosen as 800 °C (*T* *_cal_* > 700 °C).

**Figure 3:**
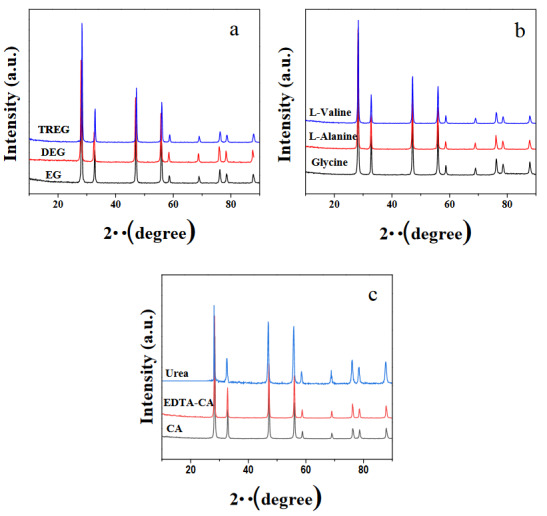
XRD patterns of calcined samples prepared with the various fuels as: a) EG, DEG, TREG b) Glycine, L-alanine, L-valine c) CA, EDTA-CA, and urea.

**Figure 4:**
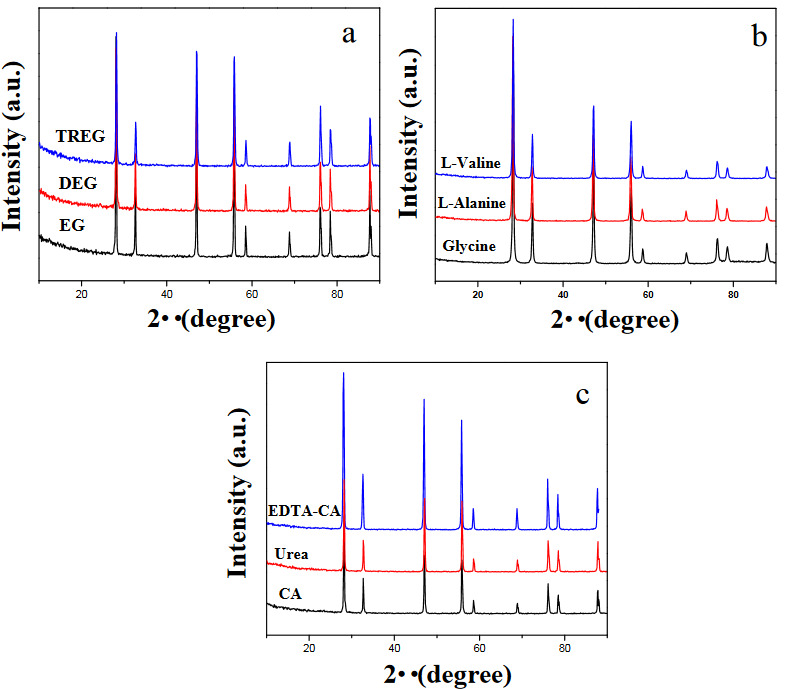
XRD patterns of sintered samples prepared with the various fuels as: a) EG, DEG, TREG b) Glycine, L-alanine, L-valine c) CA, urea, and EDTA-CA.

It can be seen in Figures 3 and 4, the X-ray diffraction (XRD) pattern of calcined and sintered SDC20 powders, respectively. In accordance with the literature, all electrolytes have only the characteristic peaks of the cubic-fluorite-type phase of ceria (JCPDS No: 81–0792) and the obtained XRD data comprises the (111), (200), (220), (311), (222), (400), (331), (420), and (333) diffraction peaks for all samples. No crystalline phase corresponding to Sm_2_O_3_ and any impurity could be found. XRD peaks became sharper and narrower with sintering treatment. As seen in Table 2, the average crystallite sizes of the sintered samples calculated by the Scherrer formula were determined between 22.1 and 29.84 nm. For the combustion methods, the crystallite size is affected by different parameters. Combustion temperature is one of them. Generally crystallite size of ceramics rises with the increase of temperature [22,23]. Combustion process are dependent on the chemical structure of fuel. Hence the crystallite size of the products is associated with the speed and temperature of the combustion process. The smallest crystallite size among glycine, L- valine, L- alanine, citric acid, urea, and EDTA fuels were determined when using urea as a fuel due to the higher burning time, as large volumes of gases evolve (such as Table 1), they increase the dissipation of heat and limit inter-particle contact. When urea is used as a fuel, the time of the ignition of the reagent and the maximum temperature increases the phase formation during the combustion with evolving of a larger amount of gases (Table 1), which is associated with the production of powders with high fraction of volume of pores, and better crystallinity [9]. The crystallite size of the sample prepared with glycine fuel is bigger than ones prepared DEG, TREG, CA, and urea as the ignition of mixture glycine-nitrate was extremely fast [24]. It is known that polyol is a chelating agent and helps bonding with ceria cations. Because of that, it can lead to higher crystallite size. Consequently, the crystallinity of SDC20 prepared with EG is higher than the DEG and TREG. In addition, Table 2 shows that the enhancement in the steric hindrance due to the chain length influence leads to the crystallite size becomes smaller. In order to increase the ionic conductivity of CeO_2_-based electrolytes, various cations (e.g. Sr^2+,^ Ca^2^, Y^3^, La^3^, Gd^3+^, and Sm^3+^) were added to the ceria crystal structure, and oxygen-ion conductivity of electrolytes was extensively studied. Also it is known that the ideal dopant for CeO_2_ structure was determined to get an effective atomic number 62 (Sm) for exhibiting lowest activation energy by density function theory [12,25]. It was determined that Sm^3+^ doped CeO_2_ exhibited the highest ionic conductivity, since the association enthalpy between the cation and oxygen vacancies in the fluorite lattice was the lowest among the ceria-based electrolytes prepared with these dopants at constant dopant concentrations [7,8]. In the literature, lattice parameters as generated from X-ray diffraction studies were found to increase when doping Sm in ceria content. While the lattice parameter value of pure CeO_2_ was 5.411Å [26], it was determined that when 20% Sm (% moles) was added to the pure ceria, the lattice parameter value increased compared to pure ceria. Although each sample contains 20% Sm by moles, the change in the lattice parameter is due to the various fuels used in the combustion method. The lattice parameters of the sintered samples were calculated by Eq.2 and Eq.3. As shown in Table 2, lattice parameters were determined between 5.436 and 5.444. 

**Table 2:** Lattice parameters and crystallite size

Fuel	Crystallite size (nm)	Lattice parameter (Å)
Glycine	26.33	5.444
L-Alanine	28.64	5.440
L-Valine	28.19	5.440
EG	27.62	5.440
DEG	23.82	5.440
TREG	22.1	5.440
CA	25.30	5.436
EDTA-CA	29.84	5.444
Urea	25.06	5.440

**Figure 5:**
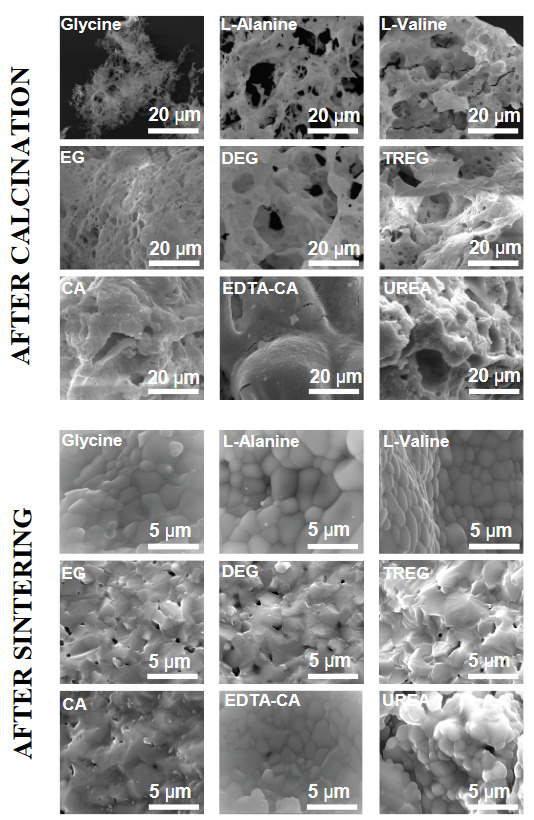
Photographs showing cross-section of SDC20 electrolytes prepared with various fuels (glycine, L-alanine, L-valine, EG, DEG, TREG, CA, EDTA-CA, and urea) calcined at 800 °C for 5h and sintered at 1400 °C for 6 h.

Figure 5 shows the SEM analysis of SDC20 electrolytes synthesized using various fuels calcined at 800 °C for 5h. All combustion agents resulted rather small and well-dispersed particles. As Figure 5 shows, SDC20 sample using glycine as fuel has extremely porous structure while SDC20 sample synthesized using L-alanine as fuel was quite dense. Electrolytes synthesized using L-valine as a fuel has a sponge-like structure. The observed porosity of amino acid fuel situation followed the order of glycine > L-alanine > L-valine. Two possible reasons may affect to the reduction of porosity: the adiabatic flame temperature and the number of the evolved gases with chain length of the fuel. Both properties are directly effective in porosity reduction. This situation has also been observed for EG-DEG-TREG analogue series. The evolved gases decrease with chain length of the fuel while adiabatic flame temperature increases. As shown in Table 1, it is determined that citric acid and EDTA-CA (mole ratio of EDTA and to citrate = 1:1) are one of good chelates and have low adiabatic flame temperature and controlled combustion reaction. Among the samples, it was found that SDC20 prepared with urea as a fuel has the lowest porosity while highest adiabatic flame temperature determined as 5854 K. It is also observed from Figure 5 that electrolytes prepared using EG, DEG and TREG, CA, and urea contain polyhedral-like shaped and very uniform spherical nanoparticles, whereas electrolytes prepared using glycine, L-alanine, and L-valine contain spherical nanoparticles. It seems that among these employed amino acid and alcohol types, it is seen that nucleation will occur instead of particle growth by increasing the steric effect. The EG sample shows higher porosity than urea, citric acid, and the other electrolytes. Also we can notice that the electrolytes prepared with urea have larger octahedral-shaped nanometric crystals than those prepared with glycine and citric acid. Therefore, these results indicate that a change in the fuel used in the synthesis lead to significant changes in the microstructure of the powders. The micrographs of the sintered samples also given in Figure 5. It is seen that all SD20 samples look comparatively dense and do not show much difference in density. It can be seen in Table 3, the average grain sizes of SDC20 electrolytes synthesized by EG are smallest and the EG shows higher porous than urea, citric acid and the other electrolytes. Another point that should be highlighted in the Figure 5 is that a change in the chemical structure of the fuel used in the synthesis leads to significant changes in the microstructure of the ceramic. 

**Table 3:** The average grain sizes of the sintered samples.

Fuel	The average grain size (µm)
Glycine	1.60
L-Alanine	2.91
L-Valine	2.25
EG	0.95
DEG	1.70
TREG	2.43
CA	1.81
EDTA-CA	1.21
Urea	1.33

**Figure 6:**
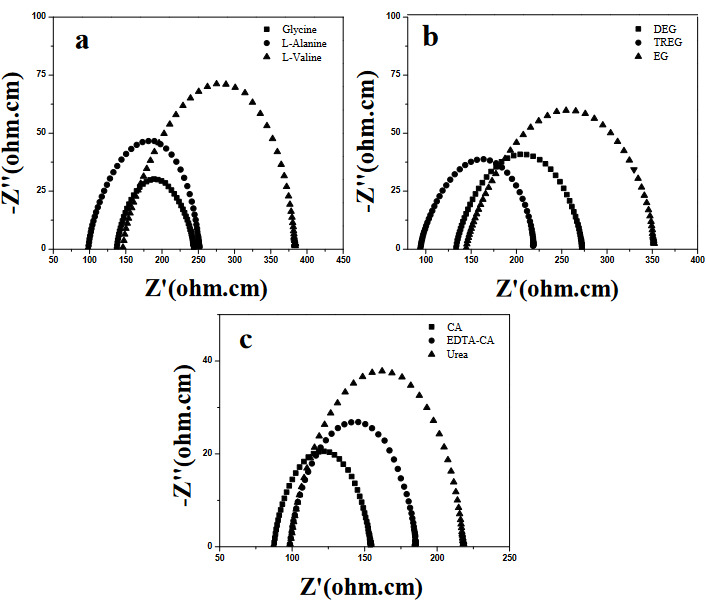
Nyquist plots of the SDC20 pellets prepared with various fuels (glycine, L-alanine, L-valine, EG, DEG, TREG, CA, EDTA-CA, and urea) at 550 °C.

Figures 6a-c show the impedance spectra of the SDC20 ceramics measured at 550 °C. The complex impedance characteristics of the SDC20 samples are similar to those of other electrolytes, such as YSZ (yttria-stabilized zirconia in the literature) [27]. At a lower temperature of 550 °C (i.e. 300 °C), the grain interior (gi), grain boundary effects (gb), and electrode polarization behavior (EPB) can be determined with three well-defined semicircular arcs. Complex impedance characteristic of the electrolytes were found similar for each sample. 

**Figure 7:**
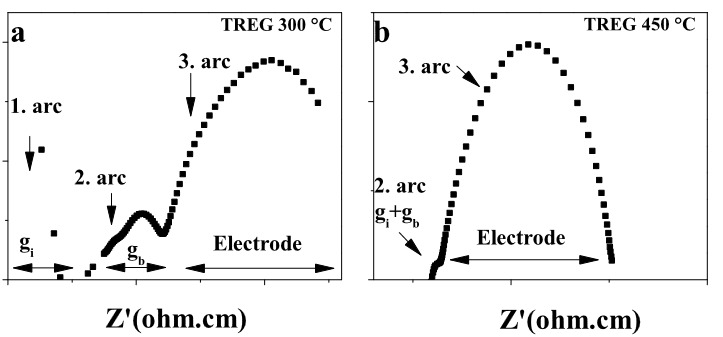
Nyquist plots of the SDC20 pellets at a) 300 °C and b) 450 °C.

**Figure 8:**
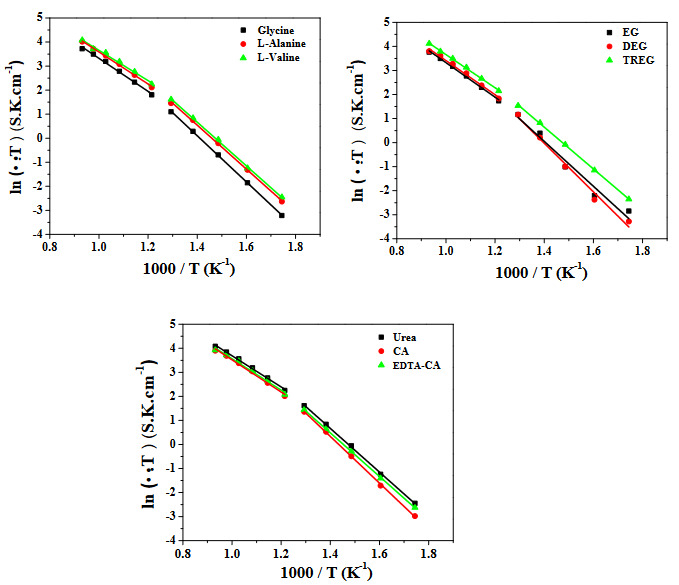
Arrhenius plots of SDC20 samples prepared with various fuels (glycine, L-alanine, L-valine, EG, DEG, TREG, CA, EDTA-CA, and urea).

To explain lower temperature behavior characteristics, the impedance spectra at low (300 °C) and intermediate (450 °C) temperature of SDC20 prepared with TREG was given in Figure 7. It is observed three arcs at 300 °C (Figure 7a), due to the bulk, grain, and electrode resistance. However, as the operating temperature increases, the semicircular arcs shift to higher frequencies, which leads to the successive disappearance of the two corresponding arcs at higher frequencies (Figure 7b and Figures 6a-c). While the circle representing the grain boundary resistances disappears at 450 °C, only the circle representing the electrode resistances is observed at 550 °C. Grain interior and grain boundary O^2−^ conductivities of the SDC20 samples sintered at 1400 °C in air atmosphere measured at 300 °C are given in Table 4. The obtained results clearly showed that bulk ionic conductivity increased with chain length of the fuel ( and (). Additionally, grain boundary ionic conductivities were lower than the grain interior ionic conductivity as expected. The ionic conductivities of the electrolytes given in Figure 6 are summarized in Table 5. The ionic conductivity of the amino acid combustion agent samples followed the order of SDC20 prepared using L-valine > L-alanine > glycine within all temperature range and the electrolyte prepared using L-valine as fuel exhibited the highest ionic conductivity. Ionic conductivity value of valine fuel situation is calculated as 5.5 x 10^–2^ Scm^–1^ at 800 °C. Tian et al. [12] analyzed sintering and ionic properties of samarium doped ceria powders fabricated with glycine-nitrate combustion. It is determined that the ionic conductivity of electrolytes increases with the sintering temperature. The ionic conductivity of 1.54 x 10^−2^ S cm^−1^ was exhibited at 600 °C for 1300 °C sintered sample. On the other hand, in this work, 2.4 x 10^−2^ S cm^−1^ O^2–^ ion conductivity measured for glycine fuel, at the same measuring temperature. For alanine situation, this value increased almost 40% and the sample performed 3.4 x 10^−2^ S cm^−1^. Also for valine fuel, this value was slightly improved to 3.5 x 10^−2^ S cm^−1^. With the current study, a 50% conductivity contribution was provided compared to the conventional glycine fuel state with the valine fuel condition. From Table 5 and Figures 8a-c, it can be seen that as the chain length of the fuel increases, the ionic conductivity increases. The same behavior was observed for glycol fuel investigations. Among the samples prepared by EG, DEG, and TREG combustion methods, the highest ionic conductivity was determined in SDC20 electrolyte synthesized with TREG-nitrate (5.72 x 10^−2^ S cm^−1^ at 800 °C). Despite our detailed search of literature, even for EG, a research study indicating the conductivity of SDC20 could not be reached. In this context, we had to compare the conductivity values of the glycol series samples with the examples found in many studies such as citric acid, urea, and glycine. For example, Wu et al. [15] discussed the conductivity performance of SDC20 synthesized by a nonion selective EDTA-citric complexing method. The ionic conductivities were strongly related to pH in EDTA-CA solution and the calcination temperature, and the electrolyte prepared with a pH of 10 exhibited the highest ionic conductivity of 1 x 10^−2^ Scm^−1^ at 700 °C and the relative density is found 99%. It was also indicated that decomposition of organic compounds is related to pH, a large amount of carboxyl groups existed in SDC20 precursor in acidic conditions and were likely to affect the sintering characteristics [15]. In this work, pH = 10 also used and ionic conductivity, relative density of the sample synthesized by EDTA-CA combustion method are 3.5 x 10^−2^ Scm^−1^ at 700 °C. This result is 250% over than that of Wu’s study. In another work Chen et al. [14] studied the effect of the urea-combustion method on the sintering and microstructure of the SDC20. It was determined that sintering temperature of 1250 °C gave a relative density of over 95%. But it is concluded that SDC20 electrolyte sintered at temperature of 1350 °C gave better total ionic conductivity with the 8.2 x 10^−2^ Scm^−1^ at 800 °C in air compared to the results of other samples. According to our study, total ionic conductivity and relative density of the electrolyte were determined as 3.35 x 10^−2^ Scm^−1^ at 800 °C and 92.11% for urea combustion synthesis. For citric acid fuel, Jaiswal et al prepared SDC20 and measured the oxygen ionic conductivity of 1.33 x 10^−2^ Scm^−1^ at 600 ºC with sintering at 1350 ºC [13]. In this study, the ionic conductivity was measured as 3.79 x 10^−2^ Scm^−1^ at 600 ºC for the same temperature. Although there is a difference between the values measured in present study and literature for the same fuels, the current situation clearly reveals the importance of this study. In this way, the importance of fuel selection can be determined more clearly. In addition, it contributes to realize the effect of different fuels on SDC20 conductivity that have not been used before in the literature. As a result, the combustion agents used for the preparation of SDC20 electrolytes exhibited the order in terms of oxygen ion conductivity measured at 800 ºC in the order of TREG > valine ≈ CA > alanine > EDTA-CA > DEG > EG > glycine > urea. 

**Table 4:** Bulk and grain ionic conductivities at 300 °C.

Fuel	gi x 10–2 (S/cm)	gb x10–2 (S/cm)
Glycine	0.00583	0.00117
L-Alanine	0.00766	0.00534
L-Valine	0.01177	0.00323
EG	0.0065	0.0045
DEG	0.0067	0.0023
TREG	0.012	0.0048
CA	0.01075	0.00425
EDTA-CA	0.0078	0.0042
Urea	0.0058	0.0025

**Table 5:** Ionic conductivities at 300 °C, 550 °C, 800 °C and activation energies of electrolytes for both low temperature and high temperature regimes.

	Total conductivity x10–2 (S/cm)			Activation energy (eV)	
Fuel	300 °C	550 °C	800 °C	Low temperature (300–500 °C)	High temperature(500–800 °C)
Glycine	0.007	0.73	3.85	0.826	0.592
L-Alanine	0.013	0.55	5.16	0.787	0.575
L-Valine	0.015	0.67	5.50	0.782	0.542
EG	0.011	0.69	4.00	0.782	0.619
DEG	0.009	0.75	4.17	0.783	0.559
TREG	0.016	1.04	5.72	0.748	0.539
CA	0.015	1.14	5.50	0.781	0.560
EDTA-CA	0.012	1.00	4.72	0.783	0.562
Urea	0.0084	1.00	3.35	0.836	0.577

Figure 8 also shows Arrhenius plots for SDC20 electrolytes. The activation energy values of the electrolytes between low (300−500 °C) and high temperature (500−800 °C) are also shared in Table 5. As can be seen in Table 5, the values of activation energy of electrolytes are in the range of 0.539−0.619 eV for the IT SOFC operation temperature interval (500−800 °C). The activation energies at low temperatures were found in the range 0.748 and 0.836 eV. It can be seen that two lines with different slopes given in Figure 8 due to change in the conduction mechanism at low and high temperature regimes [28,29]. The calculated lowest activation energy value of the sample is SDC20 prepared with TREG both at low temperature and high-temperature regimes. These results show that TREG-nitrate could be defined as novel and best combustion agent for the preparation of solid ceramic electrolyte material with the highest O^2−^ ion conductivity for SOFC applications among the nine different fuels.

## 4. Conclusion 

This work is a reference study in that it enables more clear comparison of fuels used on ionic conductivity. The objective of present investigation is to work the effect of various fuels (urea, glycine, citric acid, EDTA, TREG, DEG, EG, L-alanine, and L-valine) in the preparation of Ce_0.80_Sm_0.20_O_1.90_ (SDC20) materials by the combustion method. The combustion methods provided advantage of quickly producing fine and homogeneous powders. All the sintered electrolytes showed fluorite structure of CeO_2 _based electrolytes without impurities. Phase formation, thermal stability, and crystallite size are strongly related to the chemical structure of the fuels. The combustion agents used for the fabrication of SDC20 electrolytes exhibited the order in terms of O^2−^ ion conductivity measured at 800 ºC in the order of TREG > valine ≈ CA > alanine > EDTA-CA > DEG > EG > glycine > urea. Among synthesized SDC20 powders sintered at 1400 °C, SDC20 electrolyte prepared with TREG gave highest total ionic conductivity of 5.72 x 10^−2^ S cm^−1^ at 800 °C in air atmosphere.

## Funding

This work was supported by the Scientific and Technological Research Council of Turkey (TUBITAK) [grant number 216M509].
